# Task-constrained self-initiated attention shifts are indexed by frontal-midline theta ramping

**DOI:** 10.3389/fnhum.2025.1708257

**Published:** 2025-12-16

**Authors:** Dengzhe Hou, Sai Sun, Yasuhiro Hatori, Chia-huei Tseng, Satoshi Shioiri

**Affiliations:** 1 Graduate School of Information Sciences, Tohoku University, Sendai, Japan; 2 Research Institute of Electrical Communication, Tohoku University, Sendai, Japan; 3 Frontier Research Institute for Interdisciplinary Sciences, Tohoku University, Sendai, Japan; 4 Advanced Safety Research Group, National Institute of Occupational Safety and Health, Tokyo, Japan; 5 Interdisciplinary ICT Research Center for Cyber and Real Spaces, Tohoku University, Sendai, Japan; 6 Advanced Institute of So-Go-Chi Informatics, Tohoku University, Sendai, Japan

**Keywords:** attention shift, attentional control, EEG, frontal-midline theta, overt attention, posterior alpha, visual attention

## Abstract

In everyday vision, we often shift attention internally without external cues. These self-initiated attention shifts are fundamental to voluntary behavior but are poorly understood because most studies use cue-based paradigms that predetermine when and where to shift attention. To address this gap, we designed a multi-sequential-choice rapid serial visual presentation (RSVP) paradigm with identical visual inputs to dissociate internal and external determinants of attention across three voluntary shift types: task-constrained self-initiated, externally instructed, and unconstrained free-viewing. Participants viewed four simultaneous streams of letters and made overt attention shifts among them, while EEG was recorded. We time-locked theta (4–7 Hz) and alpha (8–12 Hz) oscillations to shift onset and found distinct signatures for each condition. Notably, a frontal-midline theta ramping was observed before self-initiated shifts but not before instructed or free-viewing shifts, suggesting a preparatory buildup of cognitive control specific to internally driven shifts. Concurrently, sustained suppression of posterior alpha occurred before self-initiated shifts. In contrast, instructed and free-viewing shifts showed relatively higher posterior alpha. These findings suggest that internally generated, goal-driven shifts engage an anticipatory frontal control mechanism indexed by theta increase and reduce posterior inhibition, whereas externally cued or unguided shifts do not. By isolating these condition-specific neural dynamics under identical external stimuli, our study identifies a unique oscillatory signature, frontal-midline theta ramping, associated with task-constrained self-initiated attention shifts.

## 1 Introduction

Selective attention is crucial for guiding human behavior, enabling us to focus on relevant sensory inputs while filtering out others. In vision, attention enhances detection and discrimination of target stimuli and biases early sensory processing (Posner, 1980; Luck et al., 1994; Desimone and Duncan, 1995; Carrasco, 2011, 2018). Understanding the neural mechanism of attentional control has been a major focus in cognitive neuroscience (Posner and Petersen, 1990; Carrasco, 2011; Petersen and Posner, 2012; Posner, 2016; Moore and Zirnsak, 2017). Studies of attention confirmed that attentional control is commonly divided into voluntary (top-down, endogenous) processes, driven by internal goals, and involuntary (bottom-up, exogenous) processes captured by salient external stimuli (Corbetta and Shulman, 2002; Xia et al., 2024).

Experimental studies of voluntary attention typically use predictive or symbolic cues to instruct observers where and/or when to attend (e.g., arrow cues in Posner’s spatial cueing paradigm) (Posner, 1980; Kashiwase et al., 2012, 2013; Shioiri et al., 2016). These designs have illuminated the neural bases of voluntary attention, but they impose artificial constraints: attention shifts are triggered by spatial or temporal cues rather than chosen freely (Müller and Rabbitt, 1989; Denison et al., 2024; Duyar et al., 2024; Lin et al., 2024). In everyday life, by contrast, people often shift attention without explicit signals. Specifically, in natural vision, one decides when and where to look based on internal goals and uncertainty. This discrepancy suggests that cue-based paradigms are over-simplified and lack ecological validity for understanding natural voluntary attention (Nadra and Mangun, 2023). Attention shifts driven by internal decisions without external cues have been called self-initiated attention shifts, referring to a subset of voluntary attention shifts (Hopfinger et al., 2010; Gmeindl et al., 2016; Wu et al., 2021, 2022). It is similar to studies of self-initiated motor/action decisions without external cues rather than the execution of saccades (Hoffstaedter et al., 2013; Khalighinejad et al., 2018). Understanding the internal decision processes that initiate attention shifts is vital for comprehending attentional control.

Previous studies have given participants freedom to choose where to attend on each trial (Taylor et al., 2008; Hopfinger et al., 2010; Bengson et al., 2014). For instance, Hopfinger et al. (2010) found that uncued self-initiated attention shifts still activated the frontal-parietal attention network but with unique hemispheric asymmetries. Bengson et al. (2014) showed that spontaneous fluctuations in occipital alpha power biased later choices of attentional focus. Rajan et al. (2019) reported that attention shift following a choice cue evoked larger frontal theta oscillations than instructed cueing, linking frontal theta to decision processes. These studies suggest that uncued, internally-driven shifts engage additional control mechanisms beyond those used for externally cued orienting.

Despite these advances, previous paradigms for studying self-initiated attention are still limited. Typically, participants choose between only two options (often just left vs. right), and they have no choice when to make a choice (e.g., by presenting a prompt) (Bengson et al., 2015, 2020). As Nadra and Mangun (2023) noted, such designs bias participants into alternating between a small set of alternatives, resembling “a soccer goalie rocking back and forth” rather than reflecting a neutral, free decision. This raises ecological validity concerns: read-world attention shifts involve many options, sequential decisions, and no explicit triggers.

To address this gap between real-world and cued attention shifts, we introduce a multi-sequential-choice rapid serial visual presentation (RSVP) paradigm that balances naturalism with experimental control. Our design presents four letter streams simultaneously, one at each screen corner, and participants shift overt attention among them. Participants can shift attention based on internal decisions or external targets, with each shift defined by eye movements. Importantly, the visual input is held constant while we vary the source of control. This allows us to compare three voluntary shift types: (1) task-constrained self-initiated shifts, where observers decide when and where to shift attention within a goal-directed task; (2) externally instructed shifts, where target presentations cue the timing and location of shifts; and (3) unconstrained free-viewing shifts, where there is no task or external cue. These shifts differ only in the source and degree of attentional control. By comparing them under identical stimuli, we can probe how internal volition versus external instruction uniquely modulates the neural dynamics of voluntary shift subclasses.

In the current study, we recorded EEG and eye movements and focused on theta and alpha oscillations time-locked to shift (saccade) onset, given their established links to attentional control (Rajan et al., 2019; Bengson et al., 2020). Frontal-midline theta (FMT) oscillations have been associated with cognitive control, decision-making, and action selection (Rajan et al., 2019), whereas posterior alpha oscillations relate to inhibitory control, spatial orienting, and sensory processing (Jensen and Mazaheri, 2010; Klimesch, 2012; Bengson et al., 2020; Nadra et al., 2023). We hypothesized that task-constrained self-initiated shifts would show distinct pre-shift neural signatures compared to externally instructed shifts, especially in FMT and posterior alpha reflecting top-down control engagement.

## 2 Materials and methods

We set up three conditions with identical stimuli but different task rules to elicit three types of attention shifts: task-constrained self-initiated (TCSI), externally instructed (EI), and unconstrained free-viewing (FV) shifts. To measure attention allocation, participants shifted attention overtly among RSVP streams, and shifts were operationalized as large saccades recorded by eye tracking.

### 2.1 Participants

Tohoku University students (age 20–30 years; 10 males, 7 females; all right-handed) participated. All had normal or corrected-to-normal vision, provided informed consent, and received compensation. The experiment took place in an electrically shielded room, with participants seated and using a chinrest to minimize movement. Two participants were excluded due to technical issues, leaving 15 participants (8 males, 7 females). This study was approved by the Ethics Committee of the Research Institute of Electrical Communication, Tohoku University.

### 2.2 Paradigm and stimuli

**FIGURE 1:**
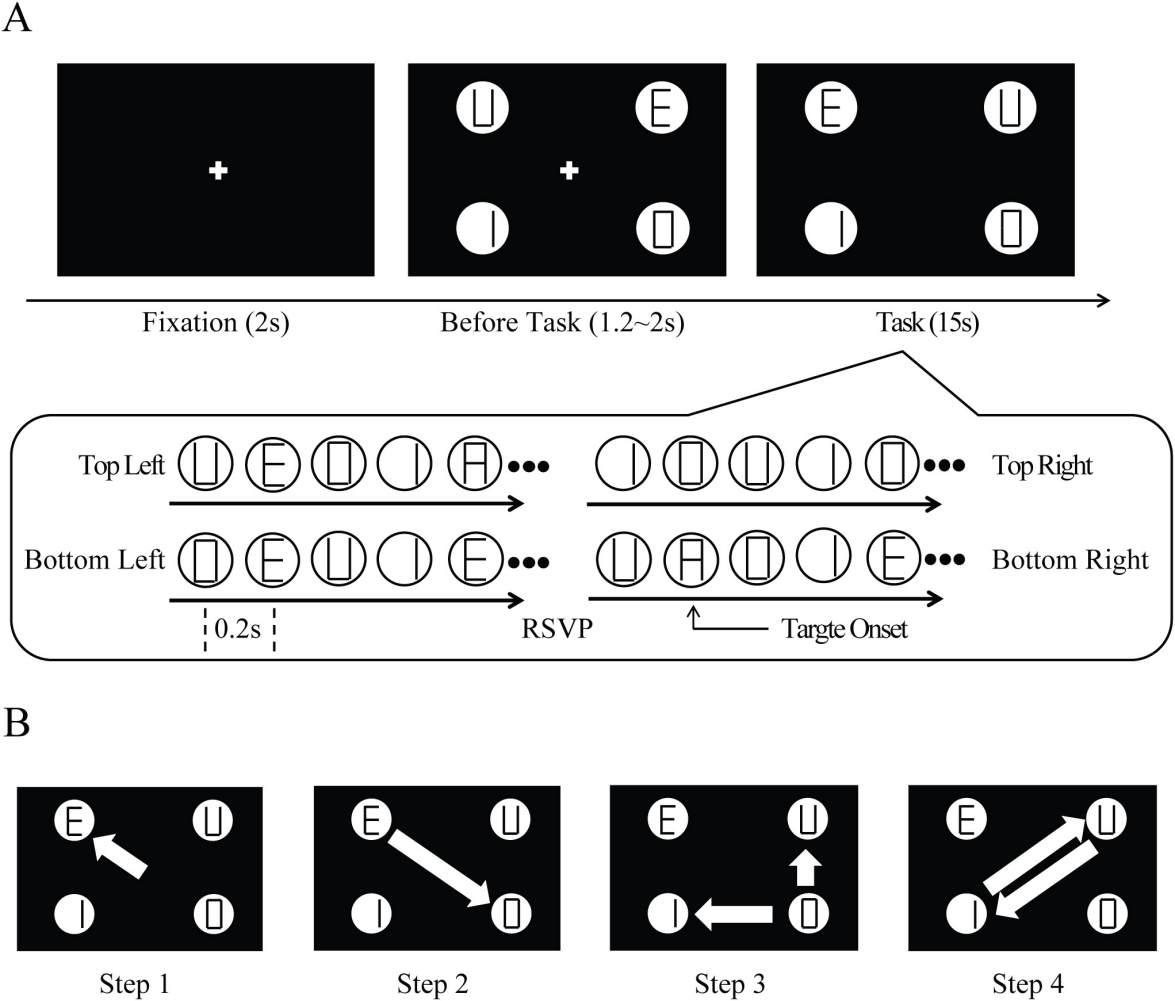
Schematic of multiple-sequential-choice RSVP paradigm. **(A)** Experimental timeline and stimulus configuration. Each trial started with a 2-s central fixation period, followed by a variable pre-trial interval (1.2 to 2 s) during which four peripheral disks containing RSVP letter sequences appeared while maintaining central fixation. The main task period lasted 15 s, and all four disks rapidly changed letter streams every 0.2 s. **(B)** Example of a prescribed shift sequence for the EI condition. This four-step protocol required participants to: (Step 1) initially direct attention to any of four peripheral disks; (Step 2) upon target detection at the first disk, shift gaze diagonally to the opposite disk; (Step 3) after identifying the target at the second disk, move attention to either of the two remaining disks; and (Step 4) complete the sequence by shifting to the final disk. White arrows indicate the direction of mandatory shifts after target detections.

Stimuli were generated in MATLAB using Psychtoolbox (Kleiner et al., 2007) and presented on a 23-inch LCD monitor (60 Hz, 1920 × 1080 resolution, viewed from ∼70 cm). The display consisted of four disks (3° in diameter) at the corners of an imaginary square, each centered 14° from a central fixation cross, at which distance. Each disk contained an independent RSVP stream: every 200 ms, one of five letters (“A,” “I,” “U,” “E,” “O”) was randomly presented (Figure 1A).

In the task-constrained self-initiated (TCSI) condition, participants performed a visual search task: they monitored the four streams of simultaneous RSVP for the target letter “A” (distractors were other vowels). The four streams were independent, and the target appeared occasionally in only one stream at a time. Each trial lasted 15 s, during which 0–2 targets could appear at 0–4 locations (mean ∼4.66 targets per trial, onset randomized ∼0.6 s after trial start). Participants reported at the end of the trial how many streams of locations (0–4) contained at least one target. No strategy could guarantee perfect answers, since monitoring all streams continuously was impossible. In practice, participants often shifted gaze between streams, deciding when to leave the current stream after some monitoring. We identified TCSI in this task by excluding any shifts immediately following a target detection, i.e., we focused on shifts made without an external cue. Since the target presentation was rare, participants often shifted gaze without detecting a target.

In the externally instructed (EI) condition, the search task was the same, but participants were instructed to shift gaze only when they detected a target (so that the target itself served as a cue). In each trial, the participant freely chose an initial stream to fixate, but the next shift was forced by the target appearance: after the first shift, three streams remained, and the second shift was to the one diagonally opposite the first (as instructed by the target), and so on (Figure 1B). Both timing and direction of shifts were determined. We analyzed the second and fourth gaze shifts in this sequence, which were driven by target cues, as the externally instructed shifts.

In the free-viewing (FV) condition, participants simply viewed the four streams freely with no task, no specified order, and no required duration at each stream. They were asked to look around naturally; any gaze shift in this condition was considered unconstrained self-initiated without a task goal.

Across conditions, each trial began when the participant pressed a key, followed by 2 s of central fixation (with the RSVP streams on-screen). After a variable pre-trial interval (1.2–2.0 s), the fixation cross disappeared and the trial task began (search or free-viewing). Trials lasted 15 s (RSVP duration), after which a query screen appeared. In TCSI and EI trials, participants responded with keys indicating which streams had targets. EEG and eye-tracking data were recorded throughout for later offline analysis.

All three conditions used identical stimuli; only the task instructions differed. This ensured any neural differences between conditions arose from differences in attentional control rather than sensory input. The experiment comprised 180 trials (30 FV, 90 TCSI, 60 EI) over 18 sessions. The condition order was always FV, then TCSI, then EI, within each block. This fixed order prevented contamination of the free-viewing strategy by knowing other tasks were coming, and prevented knowledge from earlier tasks from affecting free-viewing.

### 2.3 Eye-tracking acquisition and pre-processing

We recorded binocular eye positions at 250 Hz using a Tobii Pro Fusion system (Tobii Technology, Stockholm) simultaneously with EEG. The eye-tracker was mounted at the bottom of the screen (∼65 cm from the participants). Before each condition, we performed a nine-point calibration and validation over a 40 × 24° visual field. We analyzed the right-eye data offline in MATLAB using custom scripts. We then assessed fixation quality and cleaned the data. Trials were excluded if the initial fixation fell outside an 8 × 5° central area. We linearly interpolated any brief signal losses (e.g., blinks) between valid samples in each axis. Finally, any trials with excessive missing data or poor calibration (gaze deviating > ± 20% from center) were removed. These steps ensure precise eye-tracking input for further analysis.

### 2.4 Overt attention shift detection

We detected saccades (overt attention shift) with the velocity-acceleration-distance algorithm (Engbert and Kliegl, 2003). A saccade was identified when the gaze velocity exceeded 30°/s, and acceleration exceeded 8000°/s^2^. Gaze shifts between disks were defined as saccades larger than 10°; periods without such large saccades were treated as fixations. [Small saccades < 10° within a disk were handled by a secondary criterion; any fixation containing smaller saccades (velocity ≥ 50°/s) was also marked]. Next, we categorized each saccade by its timing relative to targets. In the visual search task, saccades occurring 0.2–1.0 s after a target onset at the current stream were labeled stimulus-driven. Saccades made without any recent target (during prolonged fixation) were labeled internally driven as TCSI.

### 2.5 EEG acquisition and pre-processing

We recorded EEG at 2048 Hz using a 64-channel BioSemi ActiveTwo system (Biosemi B.V., Amsterdam, Netherlands) with a conventional 10–20 electrode setup together with left and right mastoids for re-referencing. Analysis was performed in MATLAB using EEGLAB (Delorme and Makeig, 2004), FieldTrip (Oostenveld et al., 2011), and custom scripts. The preprocessing pipeline included: (1) downsampling to 250 Hz, (2) band-pass 0.5–40 Hz to remove slow drifts and high-frequency noise, and (3) all channels were re-referenced to the average of the two mastoid electrodes (subtracting mean mastoid activity) to reduce noise and artifact contamination. We then (4) epoched the data from 1.2 s before to 16 s after trial start, (5) ran independent component analysis (ICA) to identify and remove components corresponding to blinks, eye movements, and muscle artifacts (This procedure effectively eliminated blink and saccade artifacts during fixation, as confirmed by visual inspection of pre- and post-ICA data topographies), and (6) visually inspected the data and discarded remaining contaminated trials.

### 2.6 EEG analysis

We focused on shifts whose previous fixation durations were ≥2.5 s and the following post-fixation ≥1 s, yielding a subset of events for analysis. EEG data were segmented into 2.5-s epochs (−2.0 to +0.5 s around each saccade). We then computed frequency power using Morlet wavelets (1–40 Hz in 1-Hz steps, 4-ms time steps, 5-cycle width). For each electrode and epoch, power was baseline-normalized using the −0.5 to 0 s pre-trial interval. We extracted theta (4–7 Hz) and alpha (8–12 Hz) power time series by averaging trials within each condition. These were then averaged across participants and plotted as topographic maps at 0.2-s intervals. We quantified ramping using the linear slope of theta power, which has been widely used to capture evidence accumulation-like dynamics in neural signals (Gavenas et al., 2024). In summary, we contrasted pre-shift theta and alpha power for the three shift types (TCSI, EI, FV). Comparing TCSI vs. EI highlights neural processes unique to internally driven shifts, while TCSI vs. FV isolates task-specific effects on voluntary shifts.

## 3 Results

We first applied selection criteria to identify valid saccades (pre-fixation ≥ 2.5 s, post-fixation ≥ 1 s). This resulted in 1369 TCSI, 481 EI, and 474 FV shifts across 15 participants (mean ± SEM per participant: 91.3 ± 11.2, 32.1 ± 2.1, and 31.6 ± 4.1, respectively). We also repeated some analyses with an even stricter criterion (pre-fixation ≥ 4.5 s) to probe longer anticipatory periods. The Results below describe the key findings in a logical order: fixation durations, then spatial topographies of oscillatory power, and finally their temporal dynamics.

### 3.1 Fixation durations before shifts

**FIGURE 2:**
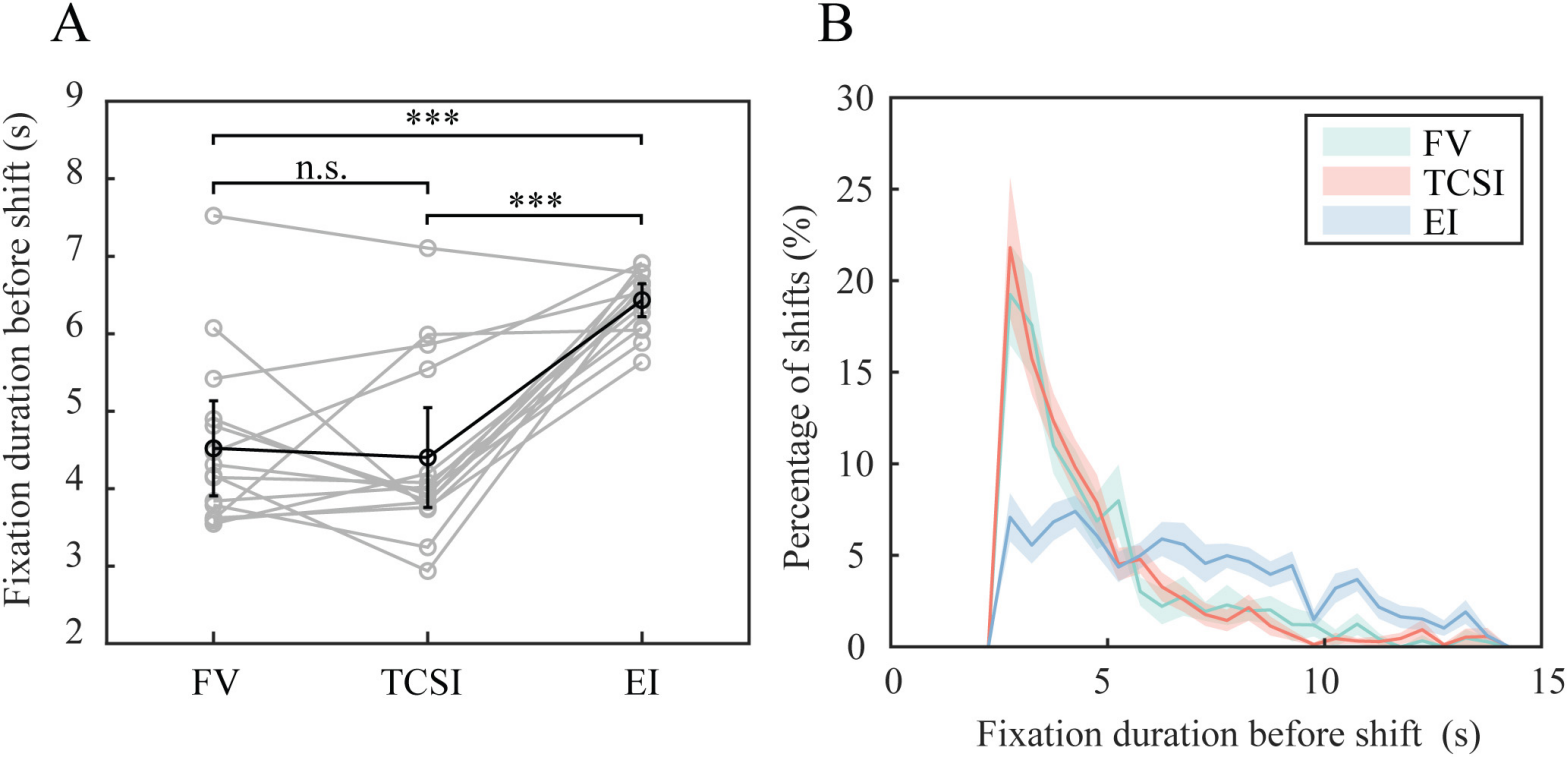
Fixation duration before shift types. **(A)** Pre-shift fixation durations. Individual data are represented by gray circles connected by gray lines to illustrate within-participant performances. Black circles and error bars indicate group means ± SEM. **(B)** Temporal distribution of shift occurrences throughout the 15-s task period. The percentage of each shift type is plotted as a function of time, with shaded regions representing SEM (***p < 0.001, n.s. *p* > 0.05).

On average, pre-shift fixation durations were 4.69 ± 1.19 s for TCSI, 6.74 ± 0.41 s for EI, and 4.57 ± 1.24 s for FV (mean ± SEM). A one-way ANOVA confirmed a highly significant effect of shift type [*F*(2,42) = 21.41, *p* < 0.001, η^2^ = 0.51]. *Post-hoc* (Bonferroni-corrected) tests showed that EI fixations were significantly longer than both TCSI (*t* = 6.68, *p* < 0.001, Cohen’s *d* = 2.35) and FV (*t* = 7.04, *p* < 0.001, *d* = 2.30). TCSI vs. FV did not differ (*t* = −0.42, *p* = 0.68, *d* = −0.10) (Figure 2A). The participants in the EI condition waited longer before moving their eyes, consistent with waiting for the target. Because saccades of EI only occurred after target presentation, the fixation durations simply mirror the target intervals in that task.

Histogram plots clarify this pattern (Figure 2B). Both TCSI and FV show a similar early peak at ∼2.75 s, indicating that people tended to shift gaze a few seconds after initial fixation (an “early engagement” with the task), followed by a rapid drop-off in shift frequency. In contrast, EI fixations are distributed more evenly over time, reflecting the unpredictable timing of targets in that condition. In other words, TCSI and FV (both self-driven) share an early surge of shifts, whereas EI (externally cued) occurs whenever the target appears. We also note that all pre-shift fixations tended to be shorter than the average target-interval duration, suggesting participants did not simply wait through the entire interval.

### 3.2 Topographies of theta and alpha bands

**FIGURE 3:**
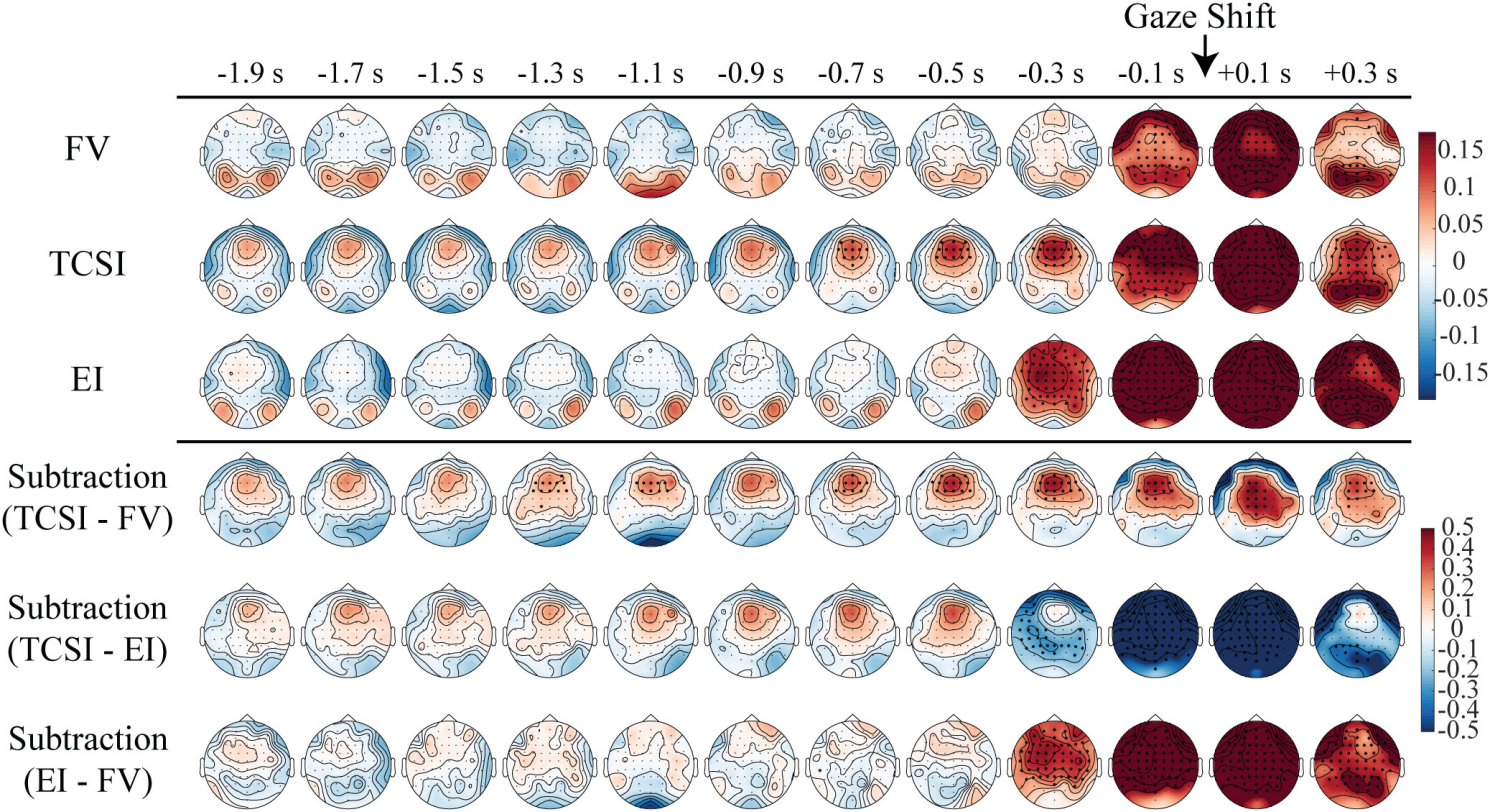
Spatiotemporal dynamics of theta power modulations before different shifts types. Topographical maps display relative theta power changes from 2 s before to 0.4 s after shift onset (0 s), sampled at 0.2 s intervals. Each row represents a shift type: FV, TCSI, and EI. The subtractions compare TCSI vs. FV, TCSI vs. EI, and EI vs. FV. Color scales represent log-transformed power values relative to baseline (–0.5 to 0 s relative to trial onset), with the upper three rows using a standardized log-power range (–0.18 to +0.18) and the subtraction rows employing an extended range (–0.5 to +0.5). Warm colors (red) indicate theta power increases, while cool colors (blue) represent power decreases relative to baseline. Black dots overlay regions showing statistically significant deviations from baseline (*p* < 0.05, cluster-based permutation test with multiple comparisons correction).

**FIGURE 4:**
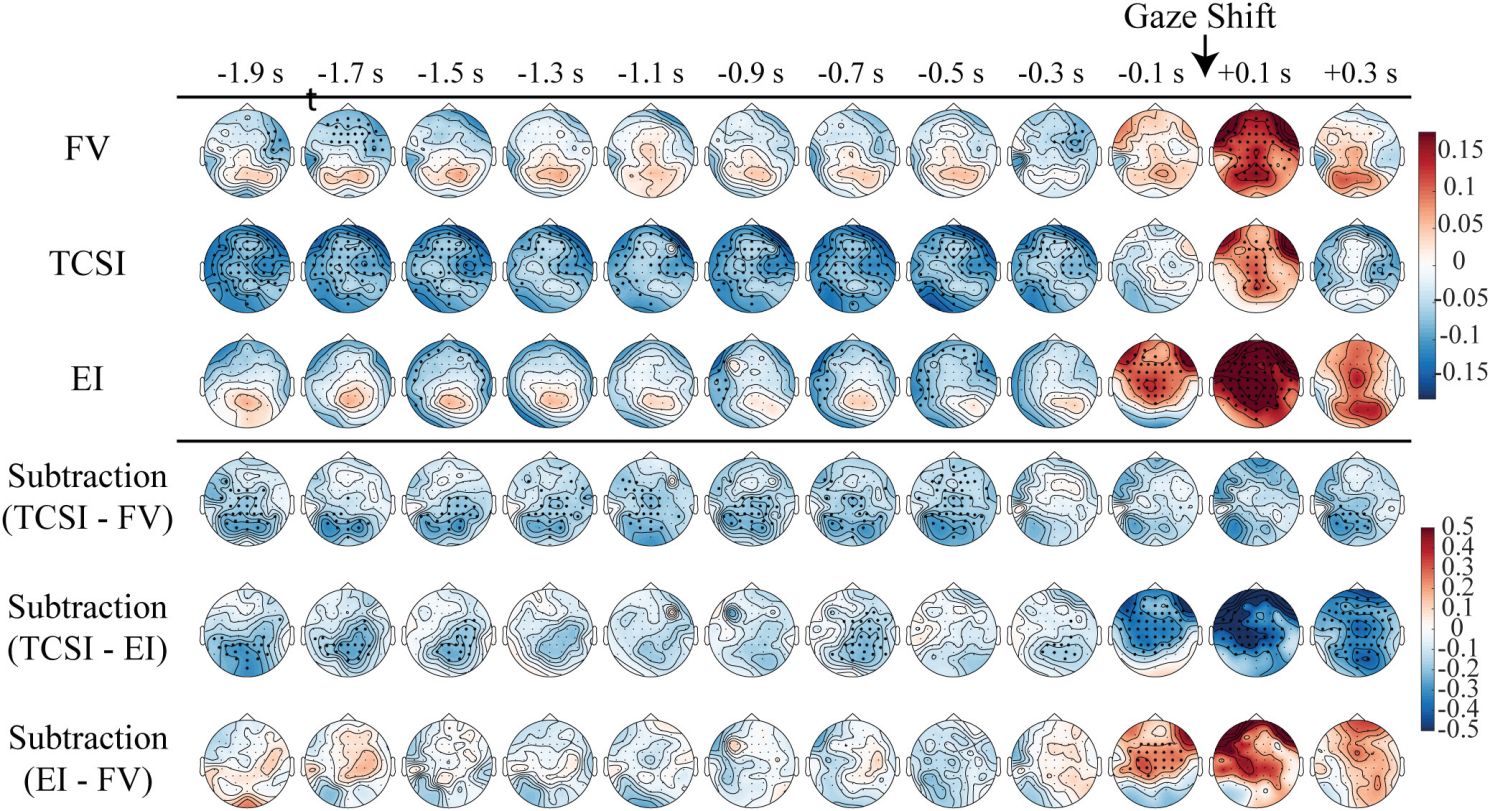
Spatiotemporal dynamics of alpha power modulations before shifts across different types. Topographical maps illustrate relative alpha power changes from 2 s before to 0.4 s after shift onset (0 s), sampled at 0.2 s intervals. The analysis encompasses FV, TCSI, and EI, with subtraction shown for TCSI vs. FV, TCSI vs. EI, and EI vs. FV. Color scaling represents log-transformed power values relative to baseline (–0.5 to 0 s relative to trial onset) with warm colors (red) indicating alpha power increases and cool colors (blue) representing power decreases. The upper three rows employ a standardized log-power range (–0.15 to +0.15), while subtraction analyses use an extended scale (–0.5 to +0.5). Black dots denote statistically significant clusters (*p* < 0.05, cluster-based permutation test with multiple comparisons correction).

Next, we examined the scalp topographies of theta and alpha power around each shift. We applied spatial cluster-based tests in 0.2-s bins rather than a single spatiotemporal cluster test, which tends to merge sustained effects into broad clusters. This temporally resolved approach allowed us to track spatial dynamics while preserving the continuous ramping pattern of theta activity. Figures 3, 4 display these maps in successive 0.2-s steps from −2 to +0.4 s relative to saccade onset, separately for TCSI, EI, and FV. Subtraction maps (bottom rows) show condition differences (TCSI - FV, TCSI - EI, EI - FV). A prominent feature in all plots is a large power surge around 0 s, present across shift types: this likely reflects generic saccade-related activity (e.g., muscle artifacts or visual disruption). Another common feature is a strong response for EI around −0.4 to −0.2 s, coinciding with target detection in that condition (since TCSI/FV have no target trigger).

Importantly, TCSI showed a distinct pattern: frontal-midline theta gradually ramped up well before the shift. Statistical testing (cluster-based permutation) confirmed that theta in frontal areas was significantly above baseline from about −0.8 s onward in TCSI (Figure 3, TCSI topographies). The difference maps highlight this: TCSI had more frontal theta and less occipital theta than FV from −2 to −0.4 s (significant for TCSI - FV). This suggests enhanced frontal control processes in TCSI that are not present in purely stimulus-driven shifts.

Alpha band topographies (Figure 4) also differ by condition. All shift types show a late surge around 0–0.2 s (again likely movement-related), but TCSI stands out in the pre-saccade interval: alpha power is generally lower (stronger suppression) in TCSI than in EI and FV. The subtraction maps show mostly negative values (TCSI - others) over parietal/occipital regions before the shift. In neural terms, lower posterior alpha is associated with increased attentional engagement. Thus, the topographical results indicate that TCSI involves a preparatory buildup of frontal theta and a sustained reduction of posterior alpha - a pattern consistent with proactive cognitive control and attention.

### 3.3 Temporal dynamics of theta and alpha bands

Finally, we quantified the time courses of the critical rhythms. We performed a participant-level bootstrap procedure (10,000 iterations) to assess EEG differences between shift types. The bootstrap procedure obtains the distribution of parameters representing features of corrected data by random sampling (Efron, 1979). Repeated resampling from data simulates new datasets, which approximate the true data distribution. One averaged slope or power was calculated over 15 times of sampling with replacement from 15 participants, and averages were compared among shift types by subtracting one from the other. Repeating the sampling and calculation provides the distribution of differences. Then we obtained distributions for three possible pairs from the same sampled dataset. If the 95% or more subtraction results are distributed in the positive region, the difference is statistically significant with 5% rejection rate. The family-wise error across the three paired contrasts was controlled using the Bonferroni correction (α = 0.05).

**FIGURE 5:**
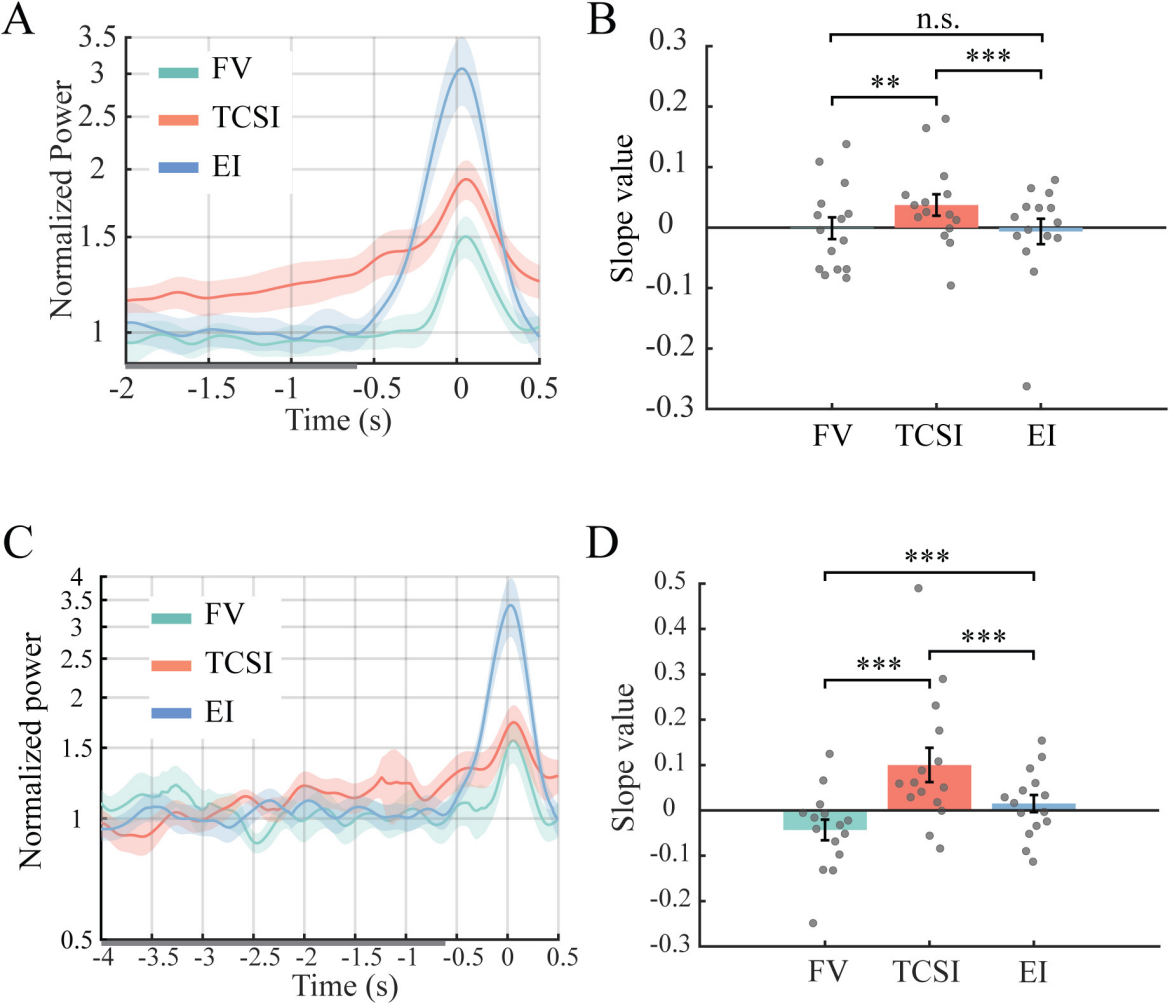
Temporal dynamics and linear trend of FMT before shifts. **(A)** Time course of normalized theta power from frontal electrodes (Fz, F1, F2) spanning 2 s before to 0.5 s after shift onset (0 s). Each panel represents a shift type: FV, TCSI, and EI. Solid lines indicate group mean normalized power with shaded regions representing SEM. The *y*-axis shows normalized power values ranging from 0.5 to 2, with 1 representing baseline activity levels. **(B)** Statistical comparison of slope values derived from individual participant linear fits during the pre-shift period (–2 to –0.6 s, gray bar). Bar plots show group mean slopes ± SEM, with statistical significance indicators above bars (**p < 0.01, ***p < 0.001, n.s. *p* > 0.05). FMT dynamics during prolonged pre-shift periods. **(C)** Time course of normalized FMT power across an extended window spanning 4 s before to 0.5 s after shift onset. Each panel represents a shift type: FV, TCSI, and EI. Solid lines indicate group mean normalized power with shaded regions representing SEM. **(D)** Statistical comparison of slope values derived from individual participant linear fits across the extended pre-shift period (–4 to –0.6 s, gray bar). Bar plots show group mean slopes ± SEM with significance indicators.

Figure 5A shows frontal-midline theta (FMT, 4–7 Hz) power (electrodes Fz, F1, F2) from −2 s to the saccade. Three frontal electrodes were selected from the regions with large power in TCSI based on observation of Figure 3. TCSI shows a gradual increase starting ∼−2 s and accelerating near −0.6 s. We fit linear slopes over −2 to −0.6 s as a window chosen to avoid any peri-saccadic effects. Figure 5B and bootstrap statistics reveal that TCSI’s FMT slope is significantly steeper than FV’s (*p* = 0.0016, corrected *p* = 0.0048, *d* = 0.55) and steeper than EI’s (*p* < 0.001, corrected *p* < 0.001, *d* = 0.58). EI vs. FV showed no significant difference (*p* = 0.64, *d* = −0.072). TCSI’s frontal theta rose much more strongly before the shift than EI and FV. These low *p*-values (<0.01) mean these differences are highly unlikely to be due to chance, and the effect sizes (d ≈ 0.55–0.58) are large by conventional standards, underscoring a robust effect.

To verify this was not a brief effect of peri-saccadic activity, we performed a variable time window analysis using the same bootstrap (Supplementary Figure 1). We used all combinations of starting and ending times between −2 to −0.6 s, while the minimum duration was set to 0.2 s, and the starting and ending times varied with a 0.1-s step. Supplementary Figure 1 confirmed the statistical differences of FMT slopes among the three shift types. The early divergences of slopes in FMT power emerged at 1.8 s (TCSI vs. FV) and 1.9 s (TCSI vs. EI) before saccade onset. Additionally, one cluster of −1.5 to −0.6 s across multiple durations shows slope divergences between TCSI and FV. While two clusters of −2 to −1.6 s and −1.3 to −0.6 s under multiple durations show slope divergences between TCSI and EI, the onset of significant divergence (∼−1.5 s) indicates that FMT ramping reflects an early preparatory control preceding TCSI rather than peri-saccadic ocular dynamics.

We also analyzed a subset with much longer fixations (>4.5 s) to extend the window back to 4 s to examine whether similar trends are seen earlier. Figures 5C, D show that even in this long-window data, TCSI had the highest theta ramping, FV the lowest, and EI intermediate; all pairwise contrasts were highly significant (*p* < 0.001). In detail, FV to TCSI (*p* < 0.001, corrected *p* < 0.001, *d* = −1.19) and to EI (*p* < 0.001, corrected *p* < 0.001, *d* = −0.72). Additionally, TCSI to EI (*p* < 0.001, corrected *p* < 0.001, *d* = 0.73). In summary, TCSI consistently exhibited the largest FMT ramping, indicating potential earlier preparatory control than 2 s.

**FIGURE 6:**
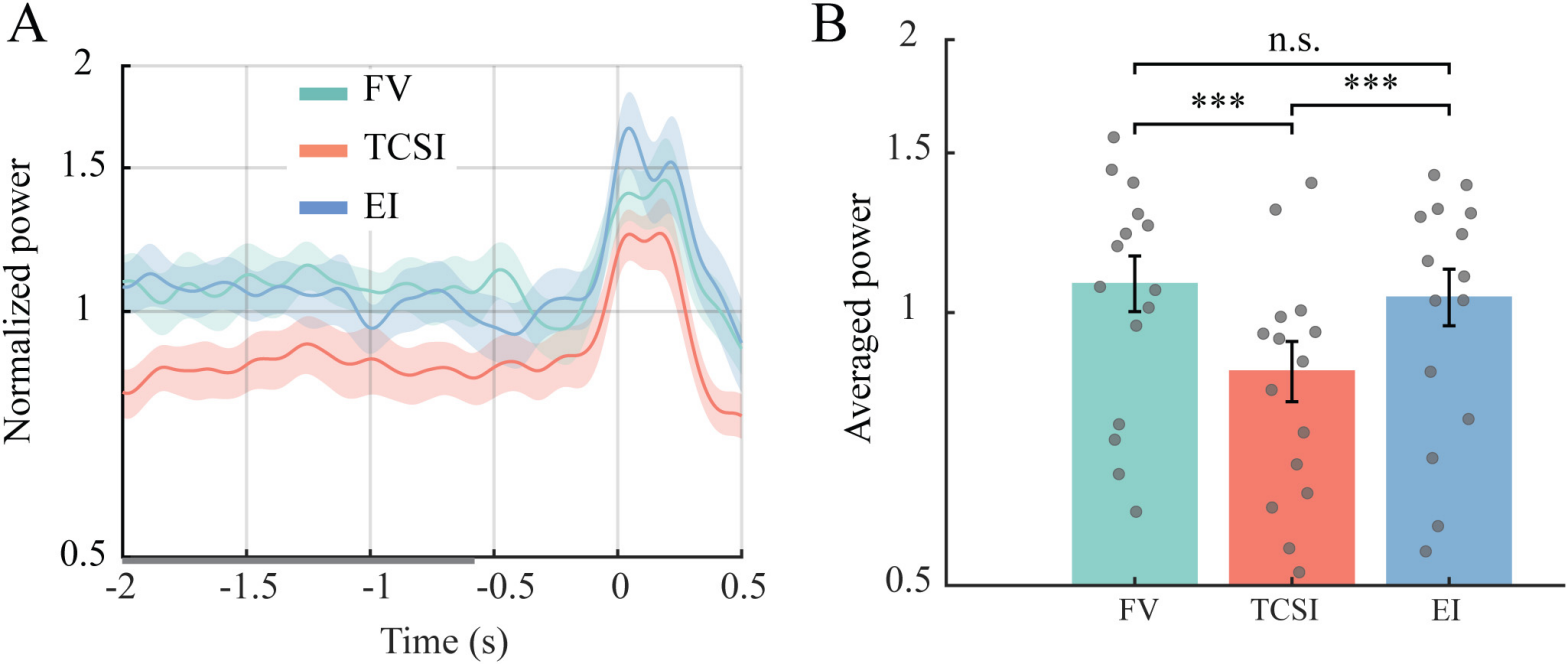
Temporal dynamics and power of posterior alpha before shifts. **(A)** Time course of normalized alpha power from posterior electrode clusters, including parietal (Pz, P1, P2), parieto-occipital (POz, PO3, PO4), and occipital (Oz, O1, O2) sites, spanning 2 s before to 0.5 s after shift onset (0 s). Each panel represents a shift type: FV, TCSI, and EI. Solid lines show group mean normalized power with shaded regions indicating SEM. The *y*-axis displays normalized power values ranging from 0.5 to 2, with 1 representing baseline activity levels. **(B)** Statistical comparison of posterior alpha power (log scale) across shift types during –2 to –0.6 s (gray bar). Bar plots display group mean normalized power ± SEM (***p < 0.001, n.s. *p* > 0.05).

We conducted analogous analyses on posterior alpha (8–12 Hz, averaged over parietal/occipital electrodes, Pz, P1, P2, POz, PO3, PO4, Oz, O1, O2), where power in EI and FV was large in Figure 4. Unlike theta, there was no pronounced pre-saccade ramping for alpha (Figure 6A). However, the average alpha level was lowest in TCSI. Bootstrap tests confirmed FV > TCSI (*p* < 0.001, corrected *p* < 0.001, *d* = 2.38) and EI > TCSI (*p* < 0.001, corrected *p* < 0.001, *d* = 2.24) with no EI vs. FV difference (*p* = 0.29, corrected *p* = 0.88, *d* = −0.44) (Figure 6B). It suggests TCSI had significantly stronger alpha suppression. Since lower alpha over visual areas reflects greater attentional allocation, this again implies that TCSI involved more active attention.

**FIGURE 7:**
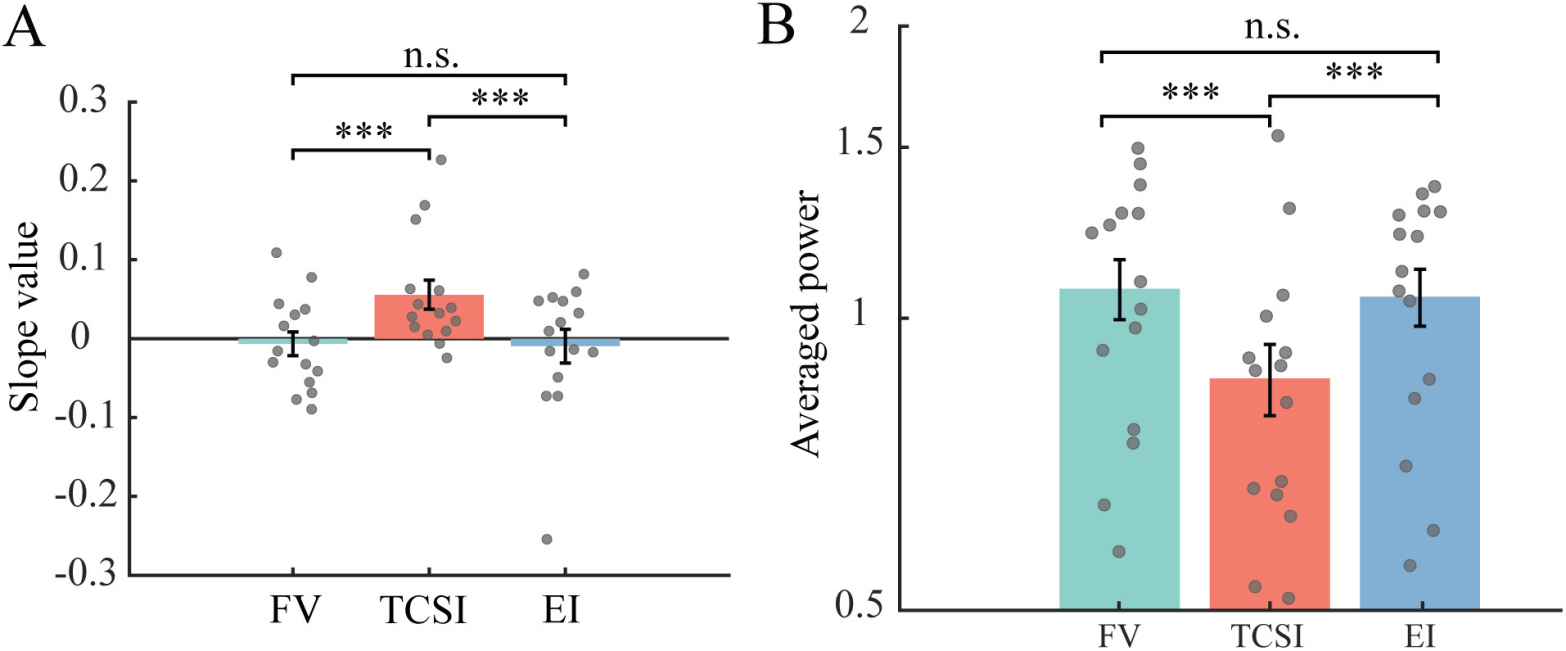
Frontal-midline theta (FMT) slope and posterior alpha power excluding small eye movements. **(A)** Statistical comparison of linear slope using FMT derived from individual participant fits during –2 to –0.6 s. Bar plots show group mean ± SEM, with statistical significance indicators above bars. Significantly steeper positive slopes of TCSI compared to both FV and EI, while FV and EI show no significant difference. **(B)** Statistical comparison of posterior alpha power (log scale) across shift types during –2 to –0.6 s. Bar plots display group mean ± SEM. Significantly reduced alpha power of TCSI compared to both FV and EI, while FV and EI exhibit comparable baseline levels (***p < 0.001, n.s. *p* > 0.05).

All results remained qualitatively the same after excluding any small saccades during fixation (removing 9.1% of shifts; 60 FV, 123 TCSI, and 29 EI): TCSI’s FMT slope was still significantly higher than FV (*p* < 0.001, corrected *p* < 0.001, *d* = 0.96) and EI (*p* < 0.001, corrected *p* < 0.001, *d* = 0.85), no significant differences were observed between EI and FV (*p* = 0.80, corrected *p* = 1.0, *d* = −0.043) (Figure 7A). The alpha level of TCSI is still significantly lower (TCSI vs. FV, *p* < 0.001, corrected *p* < 0.001, *d* = −0.71; TCSI vs. EI, *p* < 0.001, corrected *p* < 0.001, *d* = −0.66; EI vs. FV, *p* = 0.45, corrected *p* = 1, *d* = −0.07) (Figure 7B). This further confirms our findings are not artifacts of small eye movements.

## 4 Discussion

Using our multi-sequential-choice RSVP paradigm, we found distinct neural signatures among the three shift types in the cerebral cortex. Most notably, task-constrained self-initiated shifts were uniquely preceded by a sustained ramp-up of frontal-midline theta power roughly 2 s before the shift, along with a broad suppression of posterior alpha power. In contrast, externally instructed and unconstrained free-viewing shifts lacked this anticipatory theta ramping and showed relatively higher posterior alpha. Moreover, self-initiated and free-viewing shifts (both internally driven) had different neural profiles despite similar shift behavior, whereas instructed and free-viewing shifts (sharing an external cue vs. no cue) had similar neural profiles despite different task goals. These findings indicate that internally guided, goal-directed attention shifts engage a specific preparatory control mechanism that is absent when shifts are either externally cued or unguided.

To ensure our findings were not due to confounding factors, we conducted several control analyses. First, theta ramping was not attributable to behavioral differences. Pre-shift fixation durations did not differ significantly between self-initiated and free-viewing shifts (despite their different tasks) and did not correlate with the theta slope (*r* = −0.09, *p* = 0.75). Hit rates also did not correlate with theta slopes (*r* = −0.01, *p* = 0.97). This suggests that theta ramping reflects internal preparation rather than general task difficulty or motor readiness (Cavanagh and Frank, 2014). Second, the ramping was unaffected by condition order or sample size imbalance: theta slopes were stable across the first and second halves of each condition, and the results held even after resampling (100 times) to equalize shift counts. Third, we ruled out peri-saccadic eye movement artifacts: the theta divergence began around 1.8 s before the shift (Supplementary Figure 1), which is far earlier than the brief ∼0.2–0.3 s motor-related potentials (Hanning et al., 2023; Errington and Schall, 2025). Fourth, Errington and Schall (2025) showed that saccade direction can influence EEG signals several hundred milliseconds before the eye movement. However, it is unlikely that have influence of saccade direction has on theta ramping that starts much earlier (1 s or longer before the saccade) and is specific to self-initiated shifts. Moreover, the distribution of saccade directions was similar between self-initiated and free-viewing shifts, with only a mild right/down bias in instructed shifts. Finally, even after excluding trials with small fixational eye movements, the core theta ramping result remained. The above control analyses support the robustness of FMT ramping as an internal preparatory signal.

The frontal-midline theta ramping preceding self-initiated shifts likely reflects proactive engagement of internal cognitive control. Frontal-midline theta has been proposed as a signature of the need for control or decision processes in the anterior cingulate cortex (ACC) (Cavanagh and Frank, 2014; Eisma et al., 2021). Cavanagh and Frank (2014) review evidence that increases in frontal-midline theta index the accumulation of conflict or uncertainty requiring enhanced control, effectively “broadcasting” the need to adjust behavior. In our task, participants internally monitored the current stream and decided when to abandon it and shift elsewhere. The gradual theta increase in our findings suggests accumulating signals related to the decision variable in the ACC or nearby frontal regions. This is consistent with models where the ACC integrates effort, rewards, and uncertainty to trigger attention shifts when warranted (Shenhav et al., 2013). Supporting this, Rajan et al. (2019) found that voluntary attention produced increased frontal theta beginning roughly 0.4–0.8 s after a choice prompt compared to an instructed cue. Our results extend this by showing an earlier theta buildup: the ramping signal begins before the actual shift when observers decide not only where, but also when to shift (Gavenas et al., 2024). This interpretation is further supported by the evidence-accumulation-like profile of the FMT slope, consistent with recent ramping models of internally driven decisions (Schurger et al., 2012; Gavenas et al., 2024). The absence of theta ramping in instructed and free-viewing shifts suggests that when an external cue dictates a shift, or when there is no goal to achieve, this ACC-mediated preparatory control is not engaged. Overall, our findings support a mechanism in which ACC-centered theta dynamically allocates control to disengage and reorient attention when internal criteria for shifting are met.

The sustained suppression of posterior alpha before self-initiated shifts likely reflects a state of enhanced perceptual readiness. Alpha oscillations in visual cortex are strongly linked to inhibitory gating: higher alpha power corresponds to increased inhibition of that region, whereas lower alpha indicates released inhibition and greater excitability (Jensen and Mazaheri, 2010; Klimesch, 2012). Foxe and Snyder (2011) emphasize that alpha can suppress processing of irrelevant information, acting as an attentional “filter.” In our task, all four locations remained potentially relevant, but the global lower alpha before self-initiated shifts suggests a more open, exploratory mode. It may reflect a reduction in inhibitory tone, making the visual system more ready to detect targets at any location rather than selectively attending to one spot (Worden et al., 2000). In contrast, instructed and free-viewing shifts both showed relatively higher posterior alpha, implying a more conservative sensory gating. Crucially, this alpha effect cannot be explained by simple spatial attention. If alpha solely reflected selecting one visual hemifield, then self-initiated and instructed shifts (both required spatially specific monitoring) should show similar alpha. Instead, we observed a global change: a broad reduction in posterior alpha uniquely before self-initiated shifts, reflecting overall release of inhibition rather than lateralized selection.

Comparing the neural profiles of the three shift types reveals further insights. Self-initiated shifts (internally driven with a goal) evoked both frontal-midline theta ramping and posterior alpha suppression. Free-viewing shifts (internally driven without any task) showed neither: theta remained flat and alpha stayed relatively high. Externally instructed shifts (externally cued) were similar to free-viewing in oscillatory terms, despite the participants having a task in the former case. In other words, simply having an external cue - even one guiding a voluntary shift - did not engage the same preparatory control as a self-initiated decision. These patterns suggest that uncued voluntary attention may be heterogeneous: only when an internal choice is tied to a specific goal do we see the full frontal control signature. Our data suggest that task-constrained self-initiated attention recruits additional frontal systems, whereas unguided free-viewing behaves like a relatively reflexive mode. These distinctions refine models of uncued voluntary attention (Bengson et al., 2014; Rajan et al., 2019) by showing that preparatory oscillatory dynamics critically depend on contextual goals.

The multi-sequential-choice RSVP paradigm we used has clear advantages with some limitations. Its strength is in balancing experimental control with increased ecological validity. By keeping sensory input constant across conditions, we can directly compare internal versus external control of attention. Eye-tracking allowed us to mark the precise timing of attention shifts and avoid overlap between trials, addressing limitations of earlier two-choice, single-shift, cue-free tasks. Having multiple simultaneous options more closely resembles everyday attention shifts, as Nadra and Mangun (2023) suggested, “a first step is to have several possible locations to choose to attend to.” On the other hand, our setup is still artificial: the stimuli (RSVP letter streams) and the number of choices are simpler than in real scenes. Also, eye movements were overt, whereas real attention shifts can be covert. Future studies could further increase realism by using complex scenes or rewards and by removing any remaining constraints. Giving observers freedom over when to shift (not just where) is an important step toward natural behavior. Techniques like magnetoencephalography or intracranial recording could further refine the temporal resolution of these findings.

In conclusion, our results demonstrate that voluntary attention shifts driven by an internal decision recruit distinct preparatory mechanisms. Self-initiated, goal-directed attention shifts produced frontal theta buildup indexing voluntary control and a concurrent decrease in posterior inhibition indicating perceptual readiness. These markers were absent when shifts were purely externally cued or no task requirement. This suggests that goal-directed attention is a broad category, but only those shifts tied to internal objectives engage the classic volitional control network. The revealing gradual increase of theta power can be related to attentional control in ACC (Aarts and Roelofs, 2011). Taken together, our findings reveal that goal-directedness alone is not sufficient; the act of internally determining the timing of an attentional shift is what selectively engages the ACC-centered theta control network. Future work can extend this framework to more naturalistic settings, deepening our understanding of voluntary attention in real-world contexts and informing applications in brain-computer interfaces and attentional diagnostics.

## Data Availability

Publicly available datasets were analyzed in this study. Data are available from the corresponding author on reasonable request.

## Supplementary material

Supplementary Figure 1 Variable time window slope analysis of FMT. (Top) Heatmaps show mean slope subtractions across shift types for all valid start-end time window combinations between −2 and −0.6 s (minimum 0.2 s duration). (Bottom) Paired bootstrap *p*-values with colored significant cells (α = 0.05).
