# Supplementary figures and images for: Task-constrained self-initiated attention shifts are indexed by frontal-midline theta ramping
Source: Front Hum Neurosci. 2025 Dec 16;19:1708257. doi: 10.3389/fnhum.2025.1708257 (PMC12748220; doi:10.3389/fnhum.2025.1708257)

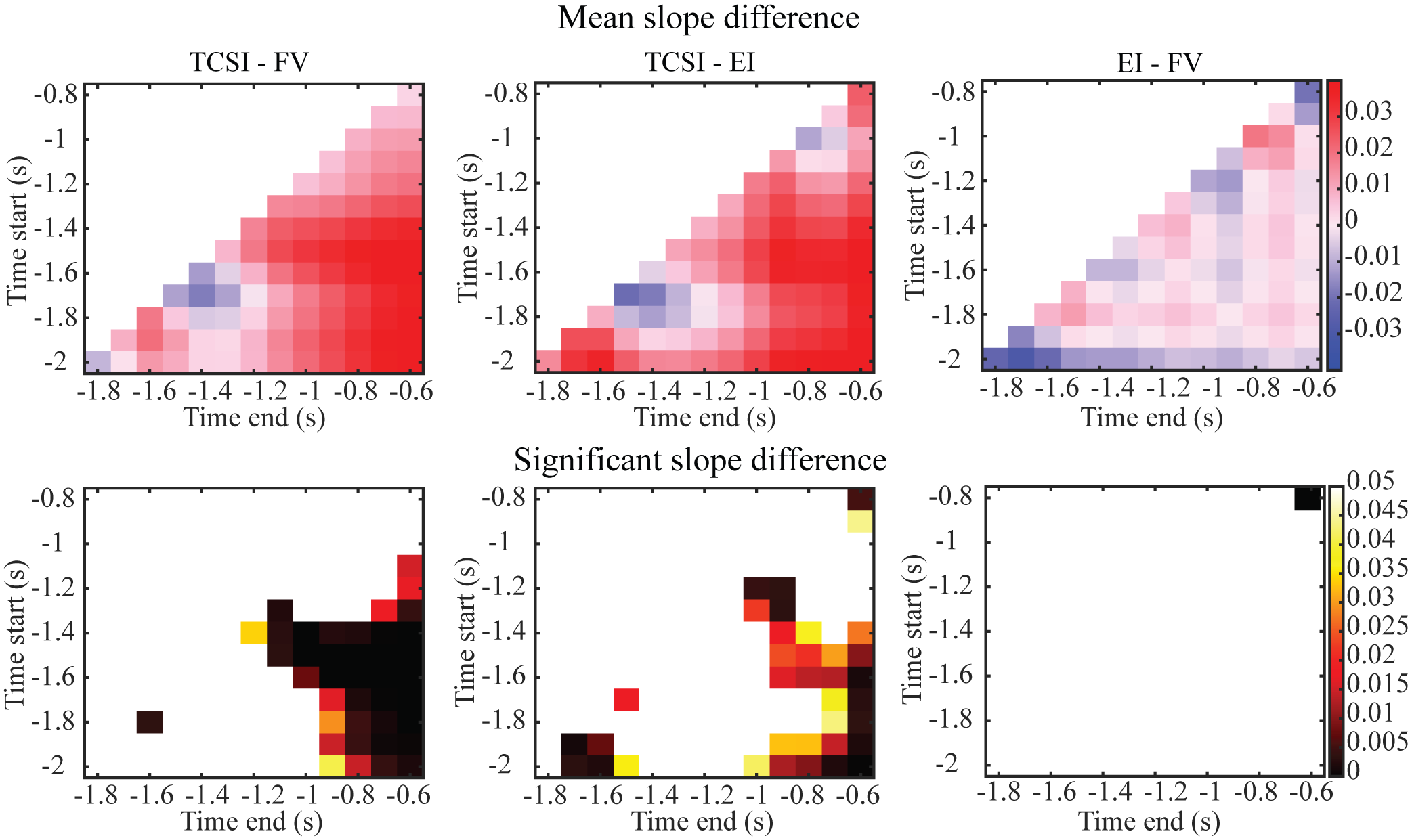

Supplement: Supplementary Figure 1 — Variable time window slope analysis of FMT. (Top) Heatmaps show mean slope subtractions across shift types for all valid start-end time window combinations between −2 and −0.6 s (minimum 0.2 s duration). (Bottom) Paired bootstrap p-values with colored significant cells (α = 0.05). [file Image_1.tif]
